# Detection and analysis of the methylation status of PTCH1 gene involved in the hedgehog signaling pathway in a human gastric cancer cell line

**DOI:** 10.3892/etm.2013.1334

**Published:** 2013-10-09

**Authors:** YUN ZUO, YU SONG

**Affiliations:** Department of Oncology, Zhangjiagang First Hospital, Zhangjiagang, Jiangsu 215600, P.R. China

**Keywords:** gastric neoplasms, tumor cell line, PTCH1 gene, DNA methylation, 5-aza-2′-deoxycytidine

## Abstract

The aim of this study was to investigate the correlation between patched 1 (PTCH1) expression and its methylation in a human gastric cancer cell line, in order to provide new information regarding carcinogenesis and the development of gastric cancer. Quantitative reverse transcription polymerase chain reaction (qPCR) and the immunocytochemical S-P method were used to identify the changes in PTCH1 mRNA and protein expression prior to and following the treatment of the AGS human gastric cancer cell line with 5-aza-2′-deoxycytidine (5-Aza-dc), a methylation inhibitor. The methylation status of the promoter region of the PTCH1 gene in the AGS gastric cancer cell line was examined using methylation-specific PCR (MSP), while CpG island methylation in the PTCH1 gene 5′ regulatory sequence was analyzed using DNA methylation analysis software. The expression of PTCH1 mRNA and protein was absent in the AGS gastric cancer cell line prior to 5-Aza-dc treatment. However, the expression of PTCH1 mRNA and protein appeared in the AGS cells treated with 5-Aza-dc. CpG island hypermethylation of the PTCH1 gene was observed in the AGS gastric cancer cell line using MSP combined with DNA sequencing. PTCH1 expression was negatively correlated with the level of promoter methylation in the AGS cells. In conclusion, the high level of methylation in the PTCH1 gene promoter region may be involved in carcinogenesis and the development of gastric cancer, and may provide a new biomarker for gastric cancer.

## Introduction

The Hedgehog (HH) signaling pathway is a crucial signal transduction pathway involved in the regulation of embryonic development. The activation of the pathway has shown a correlation with the medulloblastoma basal cell carcinoma gastrointestinal tumor and other solid tumors (1,2). Therefore, the study of the function and regulatory mechanism of the HH signaling pathway may be beneficial in the elucidation of the mechanism underlying the development of malignant tumors and in the diagnosis, prevention and treatment of tumors. The HH family mainly includes Sonic hedgehog (SHH), hedgehog interacting protein (HHIP), patched 1 (PTCH1), Smo and Gli. When SHH combines with the PTCH1 receptor, Smo is released from the combination with PTCH1 to excite Gli, prior to acting on target genes. The PTCH1 gene is one of the negative regulatory factors in the HH pathway. The suppression of PTCH1 expression is able to activate the HH pathway and is important in carcinogenesis. It has been indicated that the methylation of the PTCH1 gene may be associated with the development of certain tumors (3–5). However, the correlation between the methylation of PTCH1 and gastric cancer has rarely been explored. In the present study, the expression of PTCH1 in a human gastric cancer cell line was investigated prior to and following treatment with a methylation inhibitor, in order to explore the correlation between PTCH1 expression and the CpG island methylation of the gene promoter region. The purpose of this investigation was to understand the role of PTCH1 gene methylation in the development of gastric cancer and to provide guidance for the clinical diagnosis and treatment of gastric cancer.

## Materials and methods

### Cell line and main reagents

The AGS human gastric cancer cell line was purchased from the Cell Data Center of the Shanghai Institutes for Biological Sciences, Chinese Academy of Sciences (Shanghai, China). The DNA methyltransferase inhibitor 5-aza-2′-deoxycytidine (5-Aza-dc), a demethylation reagent, was purchased from Sigma (St. Louis, MO, USA), while TRIzol^®^ reagent was purchased from Invitrogen Life Technologies (Carlsbad, CA, USA) and the RNA reverse transcription kit was purchased from Jingmei Biological Engineering Co., Ltd. (Shanghai, China). An EZ DNA Methylation-Gold™ kit for methylation conversion was purchased from the Beijing Tianmo Technology Development Co., Ltd. (Beijing, China) and an ABI 7500 Real-Time polymerase chain reaction (PCR) instrument was obtained from Applied Biosystems (Life Technologies, Carlsbad, CA, USA). The mouse anti-human PTCH1 monoclonal antibody used in the study was purchased from Santa Cruz Biotechnology, Inc. (Santa Cruz, CA, USA), while the 3,3′-diaminobenzidine (DAB) chromogenic agent, pure xylene, hydrogen peroxide methanol, hematoxylin and neutral gum were purchased from Shanghai Ruicong laboratory equipment Co. Ltd. (Shanghai, China).

### AGS cell line culture and 5-Aza-dc treatment

The AGS cell line was inoculated in a 100 ml culture flask with a density of 3×10^5^/flask. The cells underwent conventional culture with Ham's F-12K Medium containing 10% fetal calf serum in conditions of 37°C, 5% CO_2_ for ~24 h. Once the cells had entered the logarithmic growth phase, with an 80% degree of cell fusion, the cells were treated with 5-Aza-dc (5×10^−6^ mol/l). The treatment medium was changed once every 24 h and the cells were collected 72 h later. In addition, a control group without the 5-Aza-dc treatment was cultured and collected.

### Evaluation of PTCH1 mRNA expression using quantitative PCR (qPCR)

Total RNA from the AGS gastric cancer cell line was isolated using TRIzol reagent and converted into cDNA. The primer sequences of PTCH1 were: 5′-TGT GCG CTG TCT TCC TTC TG-3′ and 5′-ACG GCA CTG AGC TTG ATT C-3′, with an amplified fragment length of 119 bp. The primer sequences of the internal control, β-actin, were : 5′-GCC ATC CTG CGT CG-3′ and 5′-TGG GCA CCG GAA CCG CT-3′, with an amplified fragment length of 260 bp. The volume of the reaction agents was 20 μl, with reaction conditions of 95°C for 5 sec, 55°C for 5 sec and 72°C for 30 sec. The PCR products were subjected to 1.5% agarose gel electrophoresis analysis.

### Evaulation of PTCH1 protein expression using immunocytochemistry

The S-P method was applied for immunocytochemistry. A 2×2 cm cover slip was soaked in concentrated sulfuric acid, autoclaved and then fixed with anticorrosive poly-l-lysine solution diluted with 1:100 ddH_2_O. The AGS gastric cancer cell line was inoculated in the sterile cover slip at a density of 2×10^4^/ml and then placed into a 90 mm petri dish. Cell specimens were extracted 3 days later and washed three times with phosphate-buffered saline (PBS). The specimens were subsequently blocked with 10% normal goat serum blocking fluid at 37°C. Twenty minutes later, the goat serum solution was disposed of. PTCH1 primary antibody working reagent (working concentration 1:100) was added into the cell specimens, prior to the specimens being incubated at 37°C. Following 60 min, the specimens were washed three times with PBS and the PTCH1 secondary antibody working solution (Shanghai Yanhui biotechnology Co., Ltd., Shanghai, China) was added at 37°C for incubation. Thirty minutes subsequently, the specimens were washed with PBS three times and stained with DAB chromogenic reagent, counterstained with hematoxylin and mounted with neutral gum. The criteria for a positive result comprised the observation of cells with brown particles in the cytoplasm and/or nucleus under a microscope.

### Assessment of CpG island methylation status of the PTCH1 gene using methylation-specific PCR (MSP) and sequencing

The DNA of the gastric cancer cell line was extracted and purified using phenol/chloroform, prior to being transformed by methylation with a methylation conversion kit. The PTCH1 MSP primers were designed using ABI Methyl Primer Express^®^ Software v1.0 (Applied Biosystems). The PTCH1 methylated primer sequences were: 5′-GTT AAT TCG TGA TTT TTC GCA-3′ and 5′-ATA ACA AAC CTA CGA ACC GC-3′, with an amplified fragment length of 197 bp, while the PTCH1 unmethylated primer sequences were: 5′-AAT GTT AAT TTG TGA TTT TTT GGA-3′ and 5′-TAA ATA ACA AAC CTA CAA ACC AC-3′, with an amplified fragment length of 197 bp. The reaction system included 5 μl 10X PCR buffer, 2 μl deoxynucleotide triphosphate (dNTP) 1, 1.5 μl methylated or unmethylated upstream primers and 1.5 μl methylated or unmethylated downstream primers, 1.0 μl TaqDNA polymerase, 32 μl ddH_2_O and 8 μl DNA converted using the methylation conversion kit. The reaction conditions were 94°C for 30 sec, 60°C for 40 sec and 72°C for 50 sec. The amplification procedure was repeated for 40 cycles, prior to the amplification products being analyzed using electrophoresis with 1.5% agarose gel. The amplified 197 bp strip was sent to the Huada Gene Institute (Beijing, China) for sequencing analysis.

## Results

### Expression of PTCH1 mRNA

**Figure 1:**
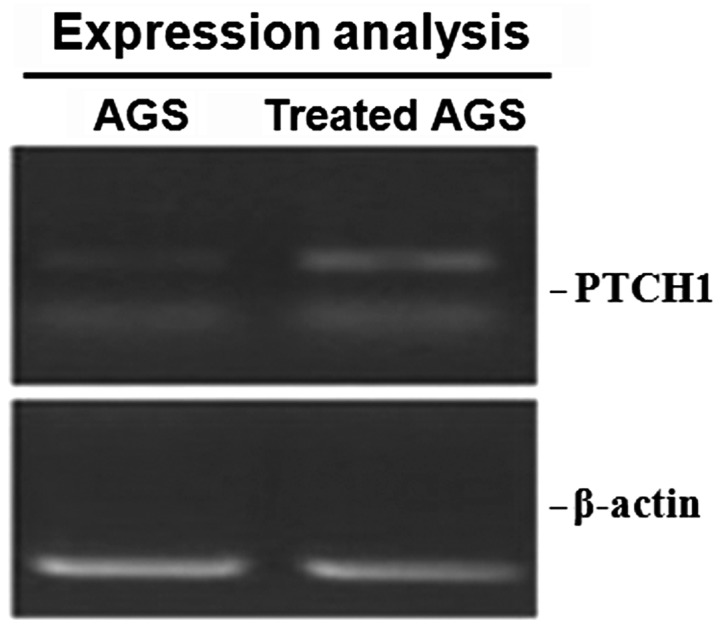
Patched 1 (PTCH1) mRNA expression in the AGS gastric cancer cell line prior to and following treatment with 5-aza-2′-deoxycytidine (5-Aza-dc).

Based on the qPCR, no amplified strip was observed in the control AGS gastric cancer cell line without the treatment of 5-Aza-dc, indicating that there was no expression of PTCH1 mRNA present. However, a 119-bp strip corresponding to PTCH1 mRNA was observed in the AGS cells following treatment with 5-Aza-dc, which demonstrated the re-expression of PTCH1 mRNA following the demethylation process (Fig. 1).

### PTCH1 protein expression

**Figure 2:**
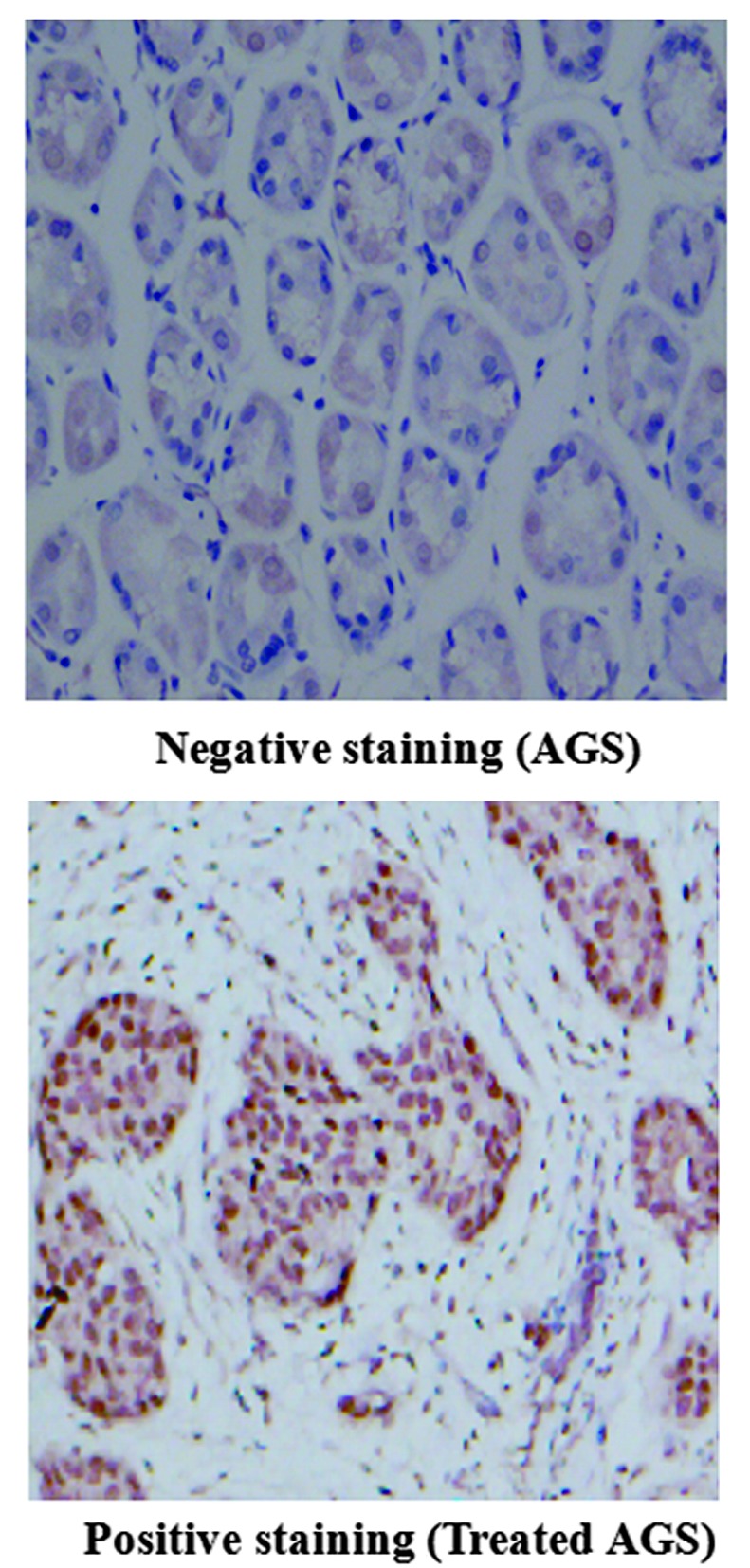
Patched 1 (PTCH1) protein expression in the AGS gastric cancer line. 3,3′-Diaminobenzidine (DAB) staining; magnification, ×400.

According to the results of the immunocytochemistry, no brown particles were observed in the AGS cells that were not treated with 5-Aza-dc, indicating that there was no expression of PTCH1 protein. Following 5-Aza-dc treatment, brown particles were observed in the AGS cells, which demonstrated the re-expression of PTCH1 protein subsequent to demethylation treatment (Fig. 2). These results were consistent with the results of the qPCR.

### CpG island methylation status of PTCH1

**Figure 3:**
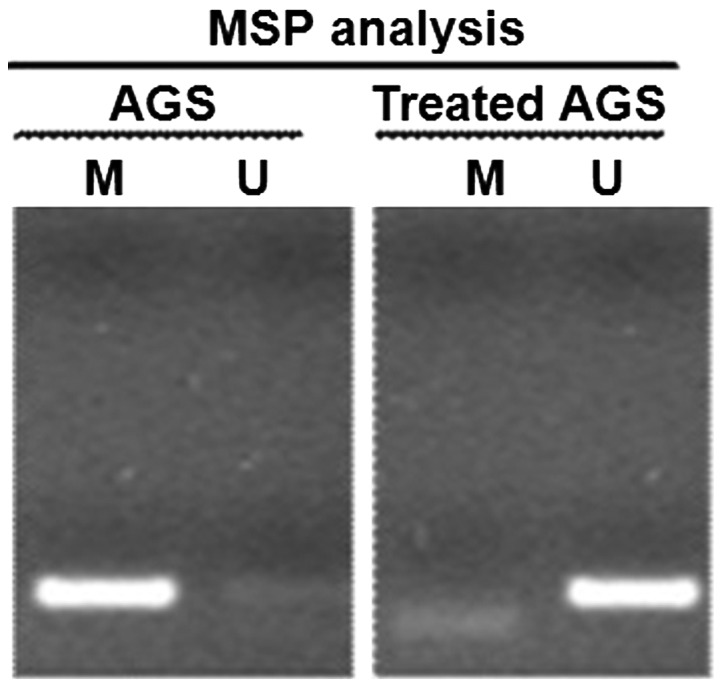
Patched 1 (PTCH1) methylation in the AGS gastric cancer cell line before and after treatment with 5-aza-2′-deoxycytidine (5-Aza-dc). M, methylated polymerase chain reaction (PCR) product; U, unmethylated PCR product.

The amplification procedure using MSP and methylated primers resulted in the presence of an amplified strip of 197 bp for the AGS cells; however, no amplified strip was observed for the AGS cells amplified using unmethylated primers. For the AGS cells that underwent demethylation treatment with 5-Aza-dc, no amplified strips were observed when the methylated primers were used; however, an amplified strip of 197 bp was observed with the use of unmethylated primers. This indicated that the PTCH1 gene of the AGS gastric cancer cell line had undergone methylation in the CpG island (Fig. 3).

**Figure 4:**
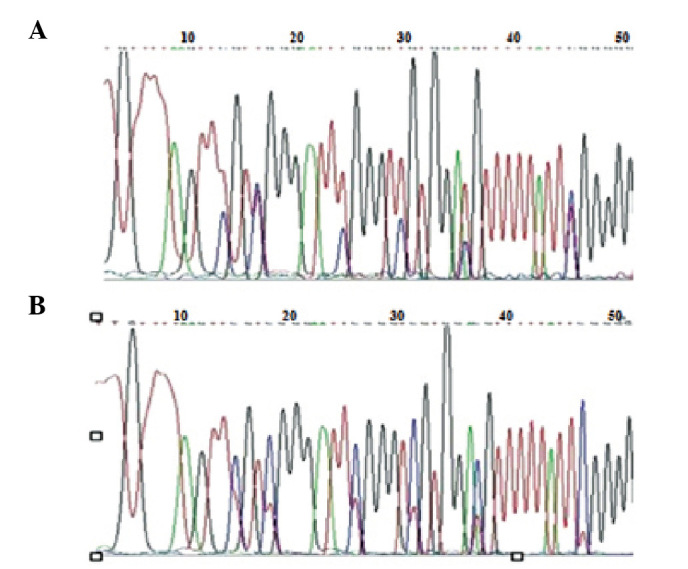
Representative figure of the methylation-specific polymerase chain reaction (MSP) sequencing analysis of patched 1 PTCH1 gene methylation in the AGS gastric cancer cell line.

**Figure 5:**
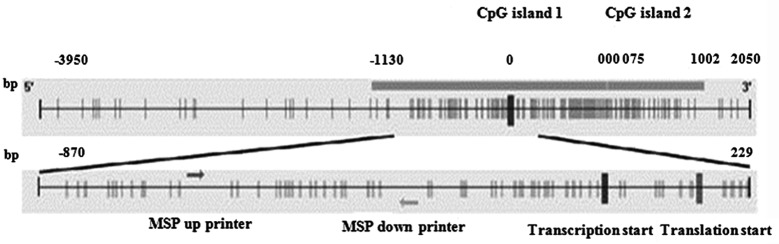
Patched 1 (PTCH1) mRNA 5′ regulatory sequence in the CpG island, as revealed by the software analysis.

According to the sequencing detection of the amplification products from the MSP with methylated primers, methylation was observed in the AGS CpG island and not in the non-CpG island region (Fig. 4). The CpG island and its location sites in the PTCH1 gene promoter region of the AGS cell line were analyzed. There were multiple transcriptional initiation sites in the PTCH1 gene. According to the analysis of the PTCH1 mRNA 1a transcriptional initiation site (as zero) using Methyl Primer Express^®^ software (Applied Biosystems), two CpG islands were apparent. One island was located between −1,139 and 860 bp and the second was located between +875 and +1,692 bp (Fig. 5). The −870 to +229 bp zone of the first CpG island was set as the target sequence and there were 19 sites in this zone. The quantitative indicator of the degree of methylation was the number of CpG sites for methylation in proportion to the total number of CpG sites detected. Statistics indicated that all methylation in the AGS gastric cancer cell line occurred in the target sequencing zone. The results of the sequencing analysis corresponded with the methylation status of the PTCH1 gene as revealed using MSP.

## Discussion

The HH signaling pathway is a critical signal transduction pathway involved in the regulation of embryonic development. HH signaling is very active in embryogenesis and then its activity disappears or reduces in normal mature tissue. However, HH signaling is extremely active in adult malignancies (6–8). PTCH1 gene is a tumor suppressor gene, located at 9q22.3, and is the most widely expressed gene in the HH family. Studies have shown that the silencing of the functional PTCH1 allele is the key factor leading to the activation of the HH pathway and carcinogenesis. The mutation of PTCH1 protein leads to the loss of its normal inhibitory function on Smo, resulting in the abnormal activation of the HH pathway and causing carcinogenesis. The expression of PTCH1 in a number of malignant tumors, such as breast, liver and esophageal cancer, has been shown to be reduced compared with that in normal tissues (9–11). In the present study, qPCR and immunocytochemical staining were used to detect PTCH1 expression in the AGS gastric cancer cell line. The results of the two detection methods were consistent. There was no expression of PTCH1 in the AGS cells that were not treated with 5-Aza-dc, whereas PTCH1 expression was observed in the AGS cells that were treated with 5-Aza-dc. This suggested a low level of PTCH1 expression in gastric cancer.

Studies on cancer epigenetics have demonstrated that there exists wide hypomethylation and some regional hypermethylation of CpG islands in the genomic DNA of tumor cells. The abnormally high methylation of the CpG island is associated with the transcriptional silencing of the gene expression and may lead to the partial inactivation of certain tumor suppressor genes. This is an important mechanism causing the malignant transformation of cells (12,13). These highly methylated genes are able to re-express gene product following the use of a demethylating agent to act on the tumor cell lines, which is important in tumor suppression (14). As such, the establishment of a DNA methylation spectral type of multiple tumor-related genes for a particular tumor, which facilitates the early diagnosis or differential diagnosis of the tumor, has been proposed (14). In a study of PTCH1 gene promoter methylation, only the regulatory sequences on mRNA 1b were assessed. The methylation analysis results for upstream regulation (−1,593 bp, with 1b transcription initiation site count) demonstrated that significantly high levels of methylation were observed in ovarian tumors; however, there were no significantly high levels of methylation in basal cell carcinoma, which retained a demethylation status (15). The methylation analysis results of the regulatory sequences (−776 to +1,238 bp, with 1b transcription initiation site count) showed that a high methylation status existed in breast cancer. The methylation level was negatively correlated with the expression of PTCH1b (9). In the present study, MSP amplification was performed to detect the CpG island methylation status of the PTCH1 gene in the AGS gastric cancer cell line. The results demonstrated that the gastric cancer cell line methylated primers amplified the corresponding-sized fragment. No appropriately-sized fragments were amplified using unmethylated primer. This indicated that the CpG island of the PTCH1 gene in the AGS cells existed in a highly methylated state. The sequences of the methylation products from MSP amplification were analyzed to investigate whether there was CpG dinucleotide methylation in the amplified fragments. This study was aimed at methylation analysis of the PTCH1a mRNA regulatory sequence. The methylation analysis of the PTCH1a upstream regulatory sequence (−870 to +229 bp, with 1a transcription initiation site count) indicated that a high methylation status existed in PTCH1 in the AGS cells, and that this high methylation status was negatively correlated with gene expression. The sequencing results were consistent with those from the MSP test. The high level of methylation of the PTCH1 gene in the gastric cancer cell line may be one of the pathogenic mechanisms of certain forms of gastric cancer. The methylation zone in the gastric cancer cell line was different from those of previous studies, indicating that the high-methylation mechanism of the PTCH1 gene promoter may be different for different types of carcinogenesis.

In conclusion, this study showed that the PTCH1 gene expression in a gastric cancer cell line was reduced. The gene expression recovered following treatment with 5-Aza-dc. MSP amplification detection and DNA sequencing showed that multiple sites of CpG island hypermethylation existed in the PTCH1 gene of the gastric cancer cell line. PTCH1 gene expression was negatively correlated with a high level of methylation. The high level of methylation in the CpG islands of the PTCH1 gene may be associated with the occurrence and development of gastric cancer. The detection of methylation of the PTCH1 gene may become a diagnostic marker of gastric cancer, which may provide guidance for the treatment and evaluation of gastric cancer.
